# Engaging Indigenous partners in health service transformation: a framework for sustained engagement built on trust

**DOI:** 10.1186/s40900-025-00721-3

**Published:** 2025-05-13

**Authors:** Carolyn M. Melro, Meghan Gilfoyle, Clifford Ballantyne, Lacey Augustine, Gregory Brass, Norma Rabbitskin, Daphne Hutt-Macleod, Srividya N. Iyer, Christopher J. Mushquash

**Affiliations:** 1 Department of Psychology, Lakehead University, Thunder Bay, ON Canada; 2 ACCESS Open Minds Indigenous Youth Mental Health and Wellness Network, Thunder Bay, Montreal, ON, QC Canada; 3 Women’s College Hospital Institute for Health System Solutions and Virtual Care, Toronto, ON Canada; 4 Douglas Mental Health University Institute, Verdun, QC Canada; 5 McGill University, Montreal, QC Canada; 6 Dilico Anishinabek Family Care, Fort William First Nation, Thunder Bay, ON Canada; 7 Thunder Bay Regional Health Sciences Centre and Thunder Bay Regional Health Research Institute, Thunder Bay, ON Canada

**Keywords:** Indigenous research, Indigenous health services, Indigenous youth, Community engagement, Mental health, Trust

## Abstract

Health research and service delivery often fail to incorporate Indigenous worldviews and local community protocols, as well as historic experiences and knowledge of harmful research practices leaving Indigenous individuals wary of participating in research. Meaningfully engaging with Indigenous stakeholders (e.g., youth, family/carers, decision-makers, and service providers) in research partnerships offers a promising pathway toward better access and quality health care and improved mental health and wellness outcomes that better meet Indigenous youths’ needs. This paper traces the development of a national research network, ACCESS Open Minds, a network of youth, family members/carers, clinicians, decision-makers and academics focused on transforming youth mental health services in Canada. The context for this network is one in which diverse Indigenous stakeholders have been engaged in health systems and service transformation against the historical and ongoing backdrop of colonialism. Within this paper, we will focus on the network’s past and on-going activities for engaging Indigenous partners to provide a critical lens on the partnership development process. We will also underscore key activities/reflections central to the development of trust and ultimately, the sustained engagement of Indigenous youth and community partners within mental health service transformation. Both trust development and sustained engagement are integral to building momentum in developing, implementing and evaluating health systems and service transformation in collaboration with Indigenous youth and community partners. We propose a framework for engaging Indigenous community partners and youth within service transformation. Trust is highlighted as the context, mechanism, and outcome. We conclude with the need to build an evidence base of what works and – and what does not work – in achieving and sustaining trust within the process of engaging Indigenous partners in health system transformation.

## Abstract (plain language summary)

Indigenous perspectives and community protocols are often overlooked in the development of health research and service delivery. Engaging in meaningful ways with Indigenous youth and community partners in research can lead to better health care access, quality, and health outcomes in health care, that are more aligned with Indigenous youths’ needs. This paper examines the development of a national research network called ACCESS Open Minds. This network includes youth, families, clinicians, community members, decision-makers, and academics, all working to transform youth mental health services in Canada. It describes how Indigenous youth and community partners have been involved in transforming health systems and services. The focus is on the network’s past and current efforts to engage Indigenous partners, providing a critical view of developing and maintain partnerships. The paper highlights key activities and reflections that are essential for building trust and sustaining engagement with Indigenous youth and community partners in mental health service transformation. Trust and sustained engagement are crucial for making progress in developing, implementing, and evaluating health systems and services in collaboration with Indigenous communities. The paper proposes a conceptual framework for involving Indigenous youth and community partners in service transformation, with trust being a central theme. It concludes with a call to build evidence on what works in achieving and maintaining trust in engaging Indigenous partners in health system transformation. Engaging Indigenous partners in health service transformation: A framework for sustained engagement built on trust.

## Background

Globally, patient and community involvement in various aspects of the research process from conception to implementation to analysis to dissemination is increasingly being seen as a necessary condition for the redesign of the health care systems and service delivery [1]. In Canada, such an approach to involving stakeholders from the outset is generally framed as “integrated knowledge translation” (IKT). An example includes the ACCESS Open Minds (ACESS-OM) network which was jointly funded in 2014 as the first Strategy of Patient-Oriented Research project by the Canadian Institute of Health Research (CIHR) and the Graham Boeckh Foundation known as Transformational Research in Adolescent Mental Health (TRAM) [2]. Although community engagement is vital to transforming the youth mental health system within Canada, engaging certain populations can require different strategies and approaches. This is especially the case for Indigenous communities as conventional research has often misrepresented Indigenous Peoples [3].

In Canada, the term “Indigenous” is commonly used by government and academic institutions as an inclusive term to refer to First Nations, Métis, and Inuit Peoples [4]. Our paper uses a distinctions-based approach by recognizing and respecting the unique rights, interests, and priorities, and concerns of different Indigenous groups and recognizing the distinct cultures, histories, rights and governance structures between and within First Nations, Inuit, and Métis populations [5]. Given the prominent role that historical mistrust and ‘helicopter’ research has had on Indigenous populations (e.g., where data is taken and never returned to community) and on-going colonial policies and practices, it is a policy and methodological imperative that Indigenous partners (e.g., youth and communities) are engaged in and lead health systems and service delivery transformation and research. Often research and service delivery fail to incorporate Indigenous worldviews and local community protocols leaving Indigenous individuals wary of participating in research. The legacy of mistreatment of Indigenous Peoples in research requires building meaningful and trusting relationships to ensure their equal partnership in the research process.

Although IKT and community-based participatory action research (CBPAR) may differ in terms of philosophical origins, they both strive to bridge the gap between knowledge and practice by promoting inclusivity, while ensuring all partners whom the research serves to benefit are actively engaged in the research process [6]. Both research approaches have been identified as underlying Indigenous Peoples’ self-determination within research in Canada [7, 8]. IKT has been described as creating safe and ethical spaces that foster trust-based relationships. It seeks to understand the context in which health service delivery and research will be implemented and requires partnerships to reflect and embrace flexibility and trust throughout the process [9]. While a breadth of research exists focusing on how to facilitate ethical, relevant, and successful Indigenous health research and research outputs [8], knowledge gaps pertaining to trust as a context, mechanism and outcome in the IKT process persist within Canada [10]. This is particularly important to explore within Indigenous contexts as Indigenous Peoples have experienced harmful research practices under the guise of advancing knowledge [11]. This has increased levels of mistrust in research, particularly when researchers are non-Indigenous or from outside the community.

Despite its importance, the challenge of defining and measuring trust persists, especially in participatory partnerships [12]. This stems from the complexities of trust as a concept [13], with an overwhelming variety of definitions associated with it [14]. This is underscored in the literature, where trust has been described as “one of the most complex, multidimensional and misunderstood concepts in the social sciences” [13](p. 117) [15–16], As discussed by Misztal et al., [17] how we understand and explore trust varies significantly by discipline, with each often drawing on their own distinct theoretical framework. Recognizing these complexities, Gilfoyle et al., 2022 [13] conducted a scoping review exploring trust conceptually and operationally in participatory research and network science literature to advance the understanding of trust in collaborative research. Indeed, a key finding from this review was the importance of conceptualizing and operationalizing trust multidimensionally, not simply as ‘present’ or ‘absent’. This was also underscored by Lucero et al., [12] who highlighted that trust research often defines and measures trust as a binary variable, ignoring its inherent complexities and dynamic nature. Further, findings by Gilfoyle et al., [13] were compatible with the realist perspective posited and enhanced by Jagosh et al., [10, 18] who emphasized that trust could operate as a context, mechanism and outcome, and as a foundational element of partnership synergy. The investigation of trust as a dynamic process remains a pressing challenge in the field of trust research [14].

To address these challenges, Gilfoyle et al., [19–21] proposed and subsequently investigated a nuanced understanding of trust, as a multidimensional model that conceptualizes trust consistently and over time, in a contextually sensitive way. Collaboration is a precondition to trust which includes seven dimensions (1. vulnerability, 2. integrity, 3. reliability, 4. shared values, visions and goals, 5. power sharing and co-ownership, 6. reciprocity and 7. ability), all of which are dynamic and influenced by partnership processes and context. Context is particularly important when building trust with Indigenous Peoples and communities as their realities are shaped by several contextual factors that will positively or negatively influence the partnership process. This includes historical and ongoing colonialism and prior experience with outside ‘helicopter’ researchers. Given this, even within broader participatory networks such as the ACCESS-OM network, it is crucial to recognize the contextual diversity of each community. While they may share common colonial histories and face similar health and social inequities, their specific circumstances will differ based on traditional worldviews, cultural practices, geographic location, governance structures, levels of self-determination, and social and economic conditions that influence their engagement within research. Communities have varying levels of community practices of engaging and participating in research, which influences the partnership and the participatory nature of the project. Some communities want to self-govern and determine the research project across all stages of the IKT cycle, while other communities welcome the support and want to provide input within choosing the topic area and design but have researchers oversee and complete the project. Researchers, funders, and ethics boards must allow for each community to decide how and to what extent they would like to be engaged in research rather than enforce prescriptive engagement approaches and frameworks.

Drawing from Gilfoyle (2024), we explore the usefulness and applicability of extending these findings at a conceptual level [13] within the context of the ACCESS-OM network while weaving in Indigenous research principles of self-determination [11, 22], cultural humility, respect, responsibility, and relevance (the additional 3 Rs of the Kirkness and Barnhardt [23]). We also extend Kirkness and Barnhardt 4 Rs (respect, responsibility, relevancy, and reciprocity) to include a 5th R, resources, based on discussions with Indigenous communities and stakeholders when describing the human and financial resources required in meaningfully creating and engaging in CBPAR. This paper describes the process of collaborating across the research cycle with the ACESS-OM network Indigenous community-partnered sites to develop a conceptual model on building and maintaining trust in different First Nations and Inuit communities.

## Methods/design

### A case to learn with: ACCESS open minds

**Fig. 1:**
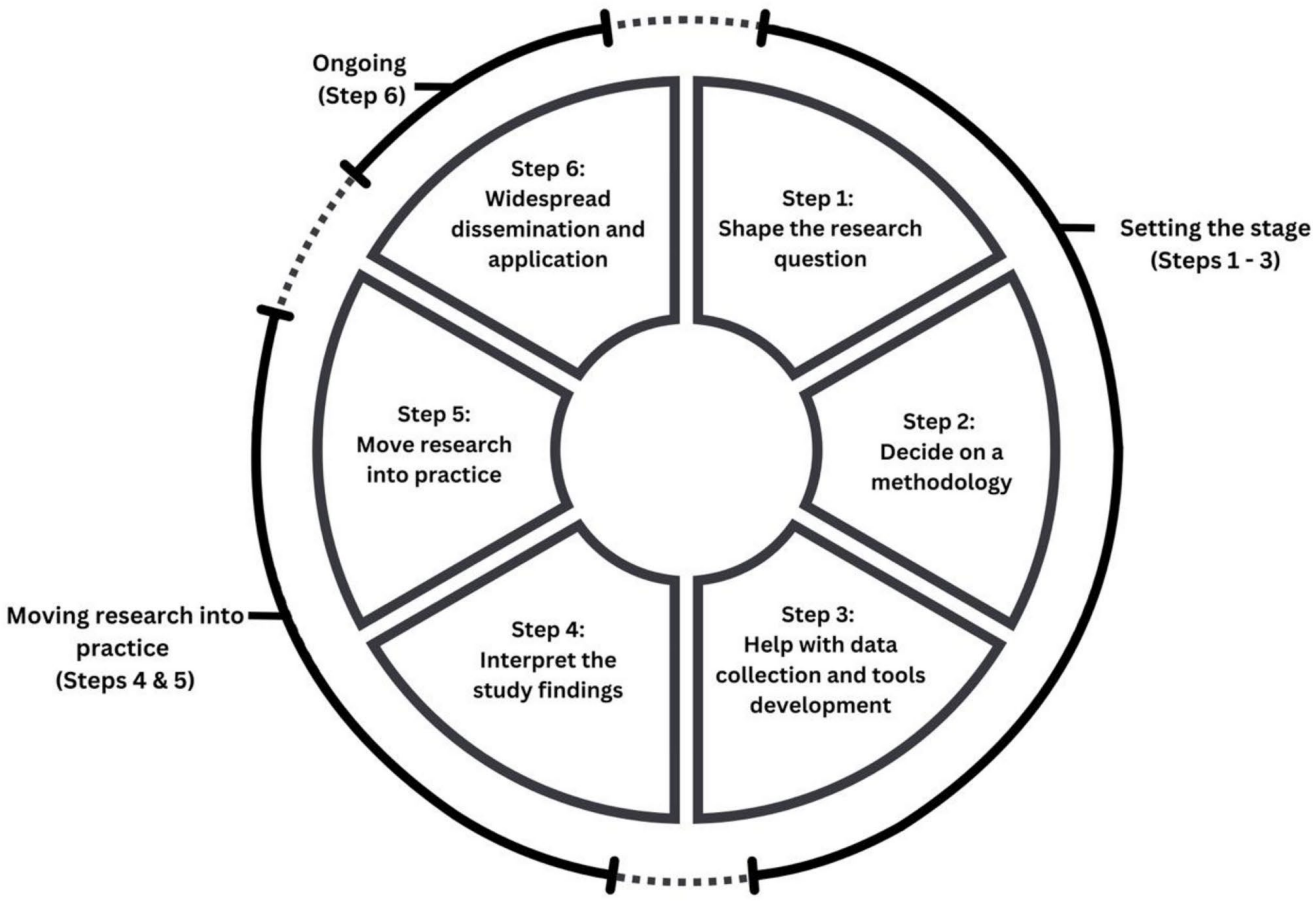
The Integrated Knowledge Translation Research Cycle (reproduced from CIHR)

The argument for Indigenous involvement in research and service transformation is clear within the literature and funding calls [24] which underscore *why* the development of strong community and youth partnerships is needed. What is missing is *how* this may be achieved. We share insights from our experience of relationships that have been built over time which led to trust with four First Nations communities: Eskasoni First Nation (Nova Scotia), Elsipogtog First Nation (New Brunswick), Aaschihkuwaataauch (Mistissini, Québec), Sturgeon Lake First Nation (Saskatchewan) and two Inuit communities Purvirnituq (Nunavik, Québec), and Ulukhaktok (Inuvialuit Settlement Region, Northwest Territories). These communities were involved in the development of CBPAR with IKT activities and processes embedded within. For simplicity, we have categorized the five steps described within the IKT research cycle identified by CIHR (See Fig. 1) into two main processes: setting the stage and moving research into practice. However, these steps are not linear and exist within a complex web of social and dynamic relations and contexts.

Before we can understand how relationships and engagement occurred within ACCESS-OM, we first need to understand its organizational structure, which has evolved as the network developed. At a network level, a backbone infrastructure, not uncommon to collective impact research, known as the central office, supports the local site operations. The network also had an executive committee, with representatives from each ACCESS-OM site, that offered guidance on operational topics such as partnerships, service transformation, and implementation of activities. The executive committee provided a crucial platform for the central office to learn from sites (e.g., yearly in-person gatherings, frequently scheduled phone conferences (at least once a month) and community visits), especially the four First Nations and two Inuit community-partnered sites. While the language and type of governance structure tends towards non-Indigenous frameworks, the value placed on the process of learning together and building shared values makes it more collaborative and less colonial. Woven throughout this paper we describe how the process of IKT facilitated trust building and sustained engagement within the ACCESS-OM network. For more information on the governance structures and shared-decision making process on the ACCESS-OM network, see Malla et al., 2019 [2], Iyer at al., 2019 [25], and Guinaudie et al., 2020 [26].

### Setting the stage (steps 1 through 3)

During the granting cycle, there were many touchpoints to co-develop the network proposal. For instance, in 2014, 55 youth, families, clinicians, researchers and decision-makers from across Canada convened in Montreal for a two-day meeting. The goal of this meeting was to collectively decide on the values and visions of the ACCESS-OM network and the necessary steps to make this vision a reality in diverse contexts. Weekly teleconferences were held after the meeting to start forming relationships and building trust through transparency and open dialogues while keeping all stakeholders engaged and collaborating in the network. Although the network was funded to focus on *all* youth mental health within Canada, the network was attuned to service transformation and evaluation needing to be community driven. For instance, in-depth site consultations after the grant was received, including with Indigenous communities, revealed that the previously conceptualized stepped-wedge cluster randomized controlled trial (RCT), would be inappropriate for Indigenous community-partnered sites, given the need for immediate resources to provide services and supports and for communities to have complete say on when to initiate services (rather than feel pressured to follow a prescribed timeline as in stepped wedge trials where sites or communities are randomized to specific times for initiating the intervention). Based on this feedback, it was collectively decided by the network that the stepped-wedge cluster RCT design would not be used network-wide. This demonstrated the network’s commitment to flexible methods to partnering with Indigenous youth and communities in service transformation and evaluation research.

In the early days of the network being operationalized, leadership at the four First Nations and two Inuit community-partnered sites advocated for self-determination over the service transformation and implementation of community-led innovations. Communities provided the ACCESS-OM network insights into historical and on-going colonialism within health service contexts. This allowed the network to better understand key gaps in youth mental health service provision and ways to improve the appropriateness and relevance of services for Indigenous youth. This was imperative to forming relationships as it required the central office staff to have the humility to reflect upon their knowledge and their understanding (or lack thereof) of historical and on-going colonialism, First Nations and Inuit community principles of research and ethics, First Nations Principles of Ownership, Control, Access and Possession (OCAP™), Inuit Qaujimajatuqangit (IQ), and Indigenous self-determination [1, 10, 22, 14, 27, 19, 28]. It was emphasized that trust needs to be bi-directional, Indigenous youth and communities trusting the researchers, and the researcher’s trusting communities in order to co-develop community services and research. This required shifting the power dynamic from the central office controlling the research to the research being self-determined by Indigenous communities in alignment with OCAP™ [19], IQ [28], and the 4 Rs framework developed by Kirkness and Barnhardt: respect, relevance, reciprocity, and responsibility [23, 15]. It required all partners to be transparent and open to learning from each other and being held accountable. When youth and a community trust that their community and cultural needs are being heard, barriers can start to break down, where the central office staff began earning communities’ trust, creating opportunities for developing better outcomes for Indigenous youth through culturally appropriate services.

Trust-building activities included key research staff from the central office planning multiple community trips throughout the duration of the project, but primarily in the early days of developing the project (e.g., data collection and tool development process, and using data to inform the development of service provision). The goal of these community visits was to learn with youth and community partners about their lived realities and to celebrate their successes once programs were launched. The network also provided funding opportunities for youth and communities to visit and learn directly from each other on their service transformation. Large network meetings were also hosted to bring together all sites for integrated knowledge sharing among the four First Nations communities and two Inuit communities, as well as informing the other non-Indigenous sites that serveice Indigenous youth in urban settings. Given geographical dispersion of communities, this also required dedicated virtual spaces for First Nations and Inuit communities to share their experiences and problem solve together. One example included 300 Eskasoni First Nation members being able to observe Puvirnituq’s (Nunavik) on-the-land healing initiative through social media.

As part of the grant proposal, a minimum evaluation protocol was implemented in various youth mental health contexts within the network to assess the effectiveness of the service transformation and refine it further based on the data being collected. As part of the service transformation, each site was provided with financial support to hire an “ACCESS Clinician”. The role of the ACCESS clinician was to ensure that youth received continuous support and had rapid access to mental health services within the community in a supportive and understanding environment. Given earlier advocacy, service transformation and evaluation looked different across the First Nations and Inuit communities to better meet their community needs and contexts. Once funding was awarded and operationalized, the ACCESS-OM central office worked with communities to develop, identify and evaluate their community-driven service transformation. This required understanding the unique but similar colonial and cultural contexts of the four First Nations and two Inuit community-partnered sites and how these contexts would influence the participation in the minimal evaluation protocol and local service transformation. For example, Ulukhaktok and Puvirnituq [29] did not follow the Minimum Evaluation protocol described because of cultural considerations, local constraints, and preferences. They preferred instead a community-driven descriptive evaluation process. They also adopted a model relying on local community workers to promote mental health literacy and wellness, and connect youth in need to supports, in lieu of hiring an “ACCESS Clinician” (a trained mental health professional). Building upon this, Puvirnituq also partnered with Saqijuq, a community-based youth diversion program given high rates of youth coming in contact with the police/justice system. In Eskasoni First Nation, their service delivery model employed Two-Eyed Seeing [30], created by honoured Mi’kmaq Elders Albert and Murdena Marshall, as a guiding principle, and which was integrated into all aspects of service delivery Within the community team in Sturgeon Lake First Nation, the model was grounded in Plains Cree cultural and language teachings [31, 32] while data collection was informed by youth. For instance, the team in the community conducted a focus group with 12 youth to discuss the minimal evaluation protocol to determine the suitability of measures being youth-friendly and culturally appropriate. Within these examples, it highlights how each youth and community had a choice in determining their participation in the service transformation and evaluation, based on their geographical and cultural context and capacity to provide specific forms of care. Within these examples, we see the “gift of multiple perspectives,” as Mushquash (2019) [33] terms it, as having great potential to inform the transformation of services in other Indigenous contexts.

### Moving research into practice (steps 4 and 5)

In alignment with community research principles and ethics, First Nations Principles of OCAP™, IQ, and Tri-council guidelines for research involving Indigenous Peoples, each of the four First Nations and two Inuit community-partnered sites, in partnership with Elders, Knowledge Holders, youth and families, participated in the interpretation of findings on service transformation and had the choice of how and if their data was to be shared. Communities took on different research and evaluation projects to show the success of their service transformation. For instance, Eskasoni First Nation and Aaschihkuwaataauch participated in a photovoice project exploring *“What does youth mental health transformation look like to you?”* OCAP™ and IQ principles were essential for creating a respectful, ethical, and effective service transformation that truly benefits Indigenous communities, and ultimately youth within communities. The network’s unique, unified minimal evaluation protocol created a data platform that brought together Indigenous services, youth, families, Elders, decision makers, as well as researchers to measure service transformation on youth’s outcomes. Leveraging this co-conceived platform, participating Indigenous communities have built capacities to gather, learn from, and share data-informed insights, and authored ten scientific publications and numerous presentations. Within this process, trust-building activities included central office staff being available to meet with community representatives to make sense of the data. It is simply not enough to send reports for community feedback, but rather researchers are required to show commitment to supporting communities’ meaningful engagement in interpreting and sharing their data. While within community, research was shared back through community events, film, and a community billboard in a highly vehicle trafficked area as determined by communities.

To inform the interpretation of findings and move research into practice, an Indigenous Council was formed. It was decided by the four First Nations and two Inuit community-partnered sites to have the Indigenous Council be co-led by an emerging Indigenous youth leader (CB). This provided one of the co-authors the opportunity to learn and build leadership and research capacity. Although the Indigenous Council was written into the TRAM funding application, members from the four First Nations and two Inuit community-partnered sites decided not to move ahead with it at the early stages of implementation. However, after some years of working alongside each other and building trusting relationships, the four First Nations and two Inuit communities decided to form the council to guide the project. The purpose of the council was to provide leadership, information, knowledge, and advice to the ACCESS-OM network and other interested parties regarding any matter affecting or pertaining to Indigenous Peoples, such as the sustainability plans for the four First Nations communities and two Inuit communities. It was expressed that the ACCESS-OM network had the responsibility of advocating for sustainability of services offered within the four First Nations and two Inuit communities to cause no harm by having services stop at the end of the five years, and to support other Indigenous communities in their efforts to improve youth mental health services by gathering and sharing wise practices to promote youth wellness, including those emerging from ACCESS-OM’s six participating Indigenous sites.

CIHR is now supporting an Indigenous Integrated Youth Services Network known as the ACCESS-OM Indigenous Youth Mental Health and Wellness Network, co-led by authors SNI and CJM (Phase 0 funded in 2022, Phase 1 funded from 2024 to 2029) [34]. The research team has also partnered in other grant initiatives (e.g., CIHR Indigenous Gender and Wellness Grants [35]; Network Catalyst Grant [36].). This was achievable through sustainable trust-based relationships formed through active, meaningful engagement and involvement of Indigenous communities in the organizational structure, and the willingness and openness of central office staff to learn with and from Indigenous Elders, Knowledge Holders, youth, and communities. Trust continues to be built and maintained with existing and new network members and communities through monthly virtual gatherings, with the option to have meetings with any Advisory Circle Member or key staff as desired. These act as a mechanism to co-develop a dynamic participatory network. As described by Jagosh et al. [10], trust building and maintenance has been an important intermediate outcome facilitating longer-term outcomes (e.g., the evolution of ACCESS-OM to the ACCESS Open Minds Indigenous Youth Mental Health and Wellness Network; building capacity among Indigenous youth and researchers who want to engage in CBPAR projects with Indigenous Peoples). This is also aligned with a study by Moore de Peralta et al. [10], who identified key participatory activities as integral for promoting trust as an outcome. Researchers bear the responsibility to understand why there may be a lack of trust by Indigenous communities in funding models and institutions given that sustainable funding has been an ongoing issue for Indigenous communities, with the need for them to re-invent themselves each time a new funding opportunity emerges. Rather, research and evaluation should support the communities’ health service transformations by exemplifying what works and to identify opportunities for growth to refine and tune their health service delivery model to meet their context and share wise practices with other Indigenous communities. The data and research collected through AOM have been tools for communities to share their stories and showcase their successes and advocate for sustainability and scalability within their community and broader integrated youth services initiatives within Canada through co-developing wise practice(s) funded in the Phase 0 project. As Provincial and Territorial IYS-networks have emerged, they are collaborating with the four First Nations and two Inuit communities to fund their service transformations and plan to expand these efforts to other Indigenous communities within their network. While this process unfortunately entailed some delays between end of funding from AOM for service delivery and commencement of other funding (e.g., from provincial integrated youth servces initiatives), the trust and relationships between the ACCESS-OM central team and the four First Nations and two Inuit communities not only persisted through these funding gaps but, against the odds, strengthened and grew. This was achieved through the ACCESS-OM network central team being transparent in the timeframe for funding and advocating for sustainable funding with communities through IKT cycles, sharing research and evaluation findings, with policy makers and funding decision makers throughout, as well as continued support as directed by communities in applying for funding through different research grants.

While the paper highlighted key activities in developing trusting relationships through the ACCESS-OM central team demonstrating trust through listening, reflecting, and acting based on communities’ needs and priorities, and following ethical and cultural practices, there were also several learnings about how trust can sometimes be disrupted, what needs to be done to navigate these situations, and how such disruptions in trust and alliance can become opportunities for clarifying values, improving process and in many ways, further strengthening bonds of trust. This is akin to the concept of productive conflicts, proposed by Jagosh et al. [10]. An example may serve to illustrate this point. About a year and a half after the grant, tensions arose between participating communities in ACCESS-OM, particularly Indigenous ones, and ACCESS-OM Central office. The central office was responding to structures (e.g., ethics committee requirements) and timelines, and the communities felt they were not heard and were not involved in decisions that were being made. It was decided that despite the pressure on the timeline, it was essential for the executive committee to meet in person. The central office also requested a well-respected Indigenous community research facilitator (not otherwise involved in the project) to facilitate the entire two-day meeting. Time was spent during the meeting in a sharing circle format to slow down and discuss the foundations of trust and each member’s best hopes from the project for their community and for how communication and relations would be in the network. This meeting helped repair ruptures in alliance and (re)build trust.

### Basket weaving: A framework for building and sustaining engagement developed through trust

**Fig. 2:**
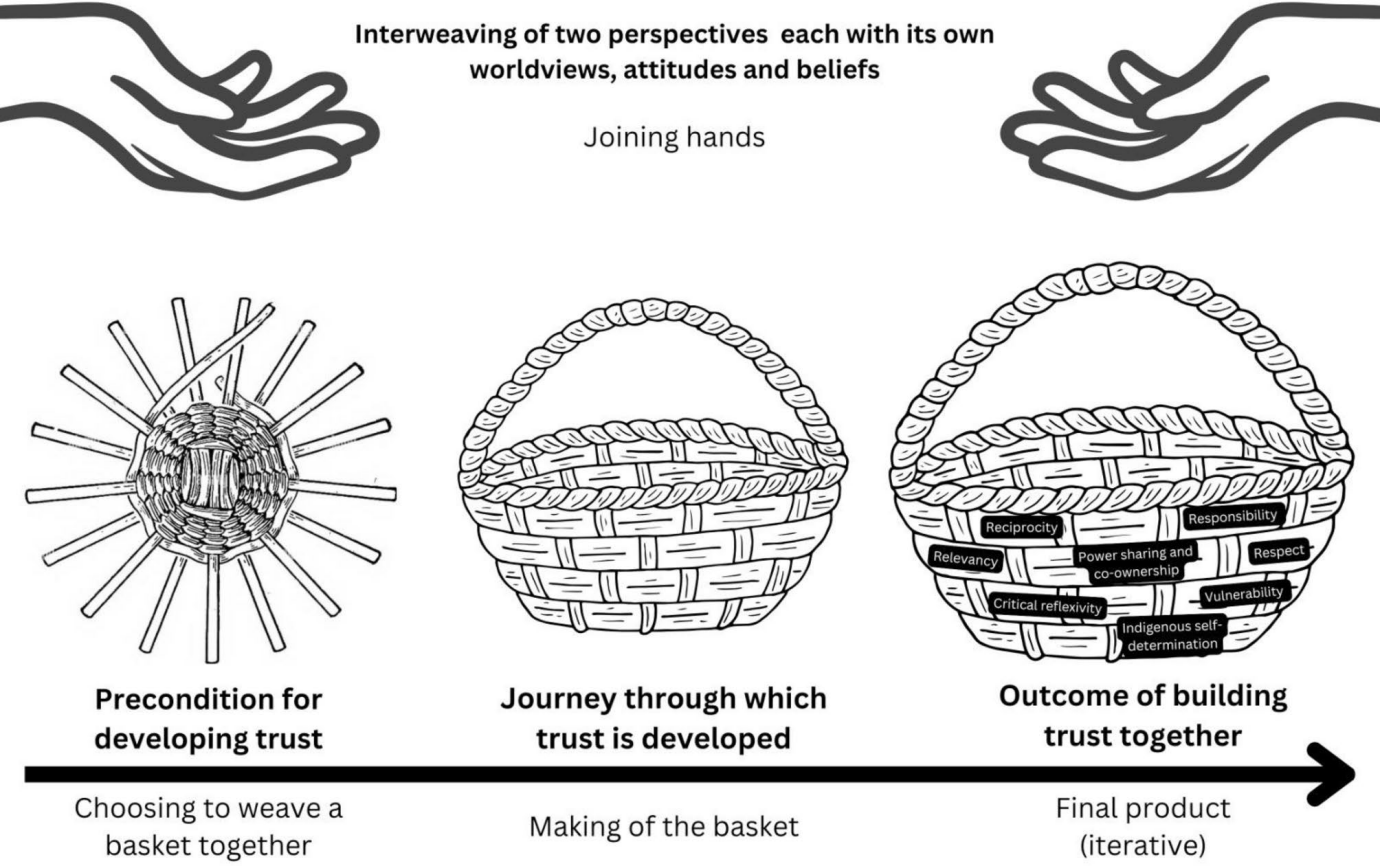
Illustration of basket weaving as a metaphor for building trust

In our experience, trust was not established within the first month, or even fully operational in the first year of the ACCESS-OM network forming. This is consistent with trust and participatory literature – trust takes time [37, 38]. We have experienced how the upfront cost of participation in CBPAR partnerships (e.g., the need for researchers to understand the historical context and power differences within a community and communities’ goals) can foster sustainable and meaningful engagement through capacity-building and trust. Applying the metaphor of basket weaving (See Fig. 2), we begin to illustrate how trust was built over time through the ACCESS-OM network and as an outcome of the process of building relationships. It is important to clarify that we do not see basket weaving as a universal cultural symbol for Indigenous populations, but rather a metaphor to illustrate coming together and being stronger through being interconnected. For instance, the act of collaborating to build a basket is the precondition to developing trust (i.e., context), making a basket is the journey through which trust is developed (i.e., mechanism), and the basket is the outcome (i.e., trust). The context in which the ACCESS-OM central office and four First Nations and two Inuit communities could come together to build a basket required the creation of a brave [39] and ethical [22] space where individuals could engage in meaningful and respectful conversations, challenging their own and other’s assumptions, through open and honest communication. This required the ACCESS-OM network to earn and demonstrate trust throughout the process of basket weaving, to draw upon the metaphor, through transparency, accountability, and following communities ethical and cultural principles requiring continued effort in engaging with communities and maintaining relationships. At times, the context in which researchers and communities initially come together is either funder-driven or shaped by funders (this was the case for ACCESS-OM with the network coming together from individuals/communities who had initially independently applied, as the call finally was set up to fund a single large network). This may result in tokenistic involvement, as well as communities perceiving involvement efforts as tokenistic. Acknowledging this risk of tokenism and power imbalances that are inherent in networks resulting from research funding and negotiating these through authentic dialogue to find shared values and objectives is at once a prerequisite to and a part of making a basket. For researchers, this negotiation process can involve a shift from viewing engagement as a requirement to viewing engagement as foundational to true service transformation. This shift, however, need not be linear; the shift may be closer to completion when the basket “trust” becomes the outcome, rather than merely a means or an instrument towards the “outcome”. In our framework, the making of a basket begins by interweaving two perspectives (as represented by the two sets of hands), each with its own worldviews, attitudes and beliefs, with one having reasonable skepticism of the other given historical mistrust among Indigenous youth and communities towards non-Indigenous researchers. Using this metaphor to view trust-building and relational development, we see this as a journey of stakeholders coming together with a **shared vision and goal** of improving Indigenous youth mental health. Similarly to IKT, basket weaving is iterative and requires materials to be flexible. Within basket weaving, materials need to be flexible so they can be easily woven. Within IKT, materials need to be relevant, understandable and meaningful to communities. This requires the basket weavers to learn which types of materials will work in which contexts, just as researchers need to learn and understand the communities’ contexts and apply a distinctions-based approach to how they partner with communities. Recognizing that no two communities are the same, just like no two baskets will be the same, we must approach each with unique understanding and respect. Metaphorically the basket has to be of use to the community, that is the research outcomes have to be of benefit to the community and the basket weavers (e.g., ACCESS-OM researchers and central staff) to continue to demonstrate trust and earn trust with communities)

With each flexible strand being woven together (the bolded words describe the strands of our proposed trust framework), a basket takes shape with an inner wall interlaced with an outer wall. The walls in our case fosters a sense of safety and belonging for Indigenous youth and communities to partner in research through culturally relevant practices, respect for Indigenous knowledge and genuine engagement with youth and communities. It is the strength between these two walls (four First Nations and two Inuit communities and ACCESS-OM researchers and staff) that give strength and purpose to the basket. If we view trust as an outcome, the basket is symbolic of trust as that outcome. Significant to this work is the openness and willingness of central office and Indigenous community partners to engage in **critical reflexivity** throughout the process via open dialogue. As part of the process, it is important to have balance all around the basket. If the central office had more power within the process, the basket would become unbalanced. This required **vulnerability** among central office staff and researchers to reflect on their research practices (e.g., learning about OCAP™ and IQ principles) and on communities’ perceived risk of losing funding if they speak up about challenges within the research governance. Through being vulnerable and engaging in open dialogue, the central office and the Indigenous sites were able to negotiate **power sharing and co-ownership** of the project that was in alignment with **Indigenous self-determination** (e.g., identify what and how data can be used). It was identified by the community that a **responsibility** of the researchers and staff was to translate data into practice in community-driven ways to ensure the service transformation was **relevant** to the community and **respects** Indigenous knowledge and cultural practices. Throughout this dynamic and complex process, **reciprocity** was demonstrated through the sites sharing community data for researchers to better advocate for the sustainability of services and supports, and researchers adapting the evaluation protocol to meet the needs of communities (e.g., using flexible methods within the service transformation and evaluation). Lastly, it is important to ensure equitable and sustainable **resources** for meaningful trust building activities (e.g., in-person meetings, longer grant calls to allow for engagement of partners, and equitable service delivery funding within Indigenous contexts) and for transfer of financial resources to communities for service transformation that meet their community needs. Drawing on the metaphor of the basket, the basket materials are only as high quality as the funding that continues to support the service transformations within communities.

## Conclusion and recommendations (discussion)

In the ACCESS-OM network, we have been working together for over 10 years to strengthen Indigenous youth mental health and wellness. Based on the established trust within the four First Nations and two Inuit communities and demonstrating trust within Indigenous communities, we have expanded our network through a kindship model, taking the early lessons learned within the ACCESS-OM network and applying it to the ACCESS-OM Indigenous Youth Mental Health and Wellness Network. We have provided key principles and values of building and sustaining trust between communities and researchers (See Table 1). The act of co-writing this current paper with community partners (listed as co-authors) is illustrative of key activities to maintaining trust, often researchers focus on publishing outcome-based studies rather than the process of how relationships and trust were developed and continue to be maintained. This is illustrative in the following quote by a co-author on the importance of these types of papers:

Reading this actually brought me to tears, not of sadness but immense happiness and pride. The way you have all written this gives validation to what our communities have been saying for generations about trust, collaboration and understanding. I honestly have no edits. I am just blown away with how powerful of a statement this is making and how I cannot wait until it is published to share with co-workers and classmates. Oelalin Nitap (thank you friend).

This article shares an early iteration of a conceptual framework for building and sustaining trust through the metaphor of basket weaving. This article builds on the work of Gilfoyle, 2023 [40] by extending their conceptual and operational model of trust in participatory research to include Indigenous self-determination, cultural humility, and respect, relevancy, responsibility, and resources. This paper uses the ACCESS-OM network context as a case to learn with. These advancements are crucial for establishing a foundation for further exploration of trust in other contexts, including future phases of the ACCESS-OM Indigenous Youth Mental Health and Wellness Network. The metaphor of basket weaving is a complex model, with several layers and elements that illustrate trust building activities and guiding principles. Similarly, the First Nations Mental Wellness Continuum Framework is grounded in culture and includes a number of elements that support health system transformation, specifically: Indigenous governance, research, workforce development, self-determination, and performance measurement of service transformation. Future research within our network work is to use the First Nations Mental Wellness Continuum framework as an evaluation tool on how trust is developed and sustained in our participatory network by measuring whether our network fosters spaces of hope, meaning, purpose and belonging.

**Table 1:** Recommendations related to principles and values of Building and sustaining trust

Recommendations	Description	Example within our network
Shared values, vision and goals	Deciding on common goals, missions, values and plans within a partnership can help to promote trust. Establishing these shared values, vision and goals at the outset of the partnership is key, ensuring all partners are “on the same page”. Checking in with these values, vision and goals as the partnership evolves can help to ensure trust is being built and/or maintained.	Collectively working towards enhancing Indigenous youth mental health and wellness though community-led innovations.
Power sharing and co-ownership	Decide collaboratively how decision-making, roles, and resources are shared within the partnership. Consider who the voices are around the table, and how these voices can be amplified. Ensure a shared understanding of ownership (e.g., of data, knowledge outputs) at the outset of the partnership. Building trust is possible when power is shared and there is co-ownership of the research decisions, processes, and outputs.	Forming and implementing the Indigenous Council to guide the network on integrating Indigenous knowledge into research and service transformation. This also included having Indigenous youth and family representatives alongside researchers, service providers, decision makers within the governance structure.
Vulnerability	Work towards an acceptable level of vulnerability through ownership of actions and ensuring transparency with all partners. Trust is built by understanding and accepting a certain level of risk and uncertainty when working collaboratively in partnership. As trust is built, this risk and uncertainty diminish over time	Contracting an external First Nations facilitator to (re)build trust through facilitating sharing circles to identify how Indigenous community-partnered sites feel their community partnership is being valued and honored (or not) and how the ACCESS-OM central team was being responsive to the diverse needs and aspirations of all Indigenous stakeholders across the four First Nations and two Inuit communities.
Critical reflexivity	Explore your own experiences, beliefs, and knowledge with honesty and humility in order to recognize our own biases, assumptions, and reactions towards Indigenous Peoples. Trust is built through this self-awareness where we acknowledge mishaps and implement changes based on these lessons learned and ensure we follow up to ensure we have heard and addressed the disagreement.	An ongoing embedded action and responsibility to ensure that the ACCESS-OM central office team was reflecting on how their positionality - such as their professional role, cultural background, or experiences—affects their interactions with Indigenous partners; and how they are (un)learning how historical and on-going colonialism continues to shape societal structures, systems and beliefs.
Self-determination	Understand and learn the communities’ cultural protocols and allow and support communities to determine their research. Trust is built when communities feel valued in that their cultural protocols and local contexts are taken into consideration within the research process.	Within ACCESS-OM, the service transformation was tailored to each of the four First Nations and two Inuit community-partnered sites. This included the “ACCESS Clinician” being different within communities to meet their context and need. All sites also included natural helpers within the service transformation as they play vital roles in supporting community well-being.
Respect	Respect community knowledge and expertise by intentionally listening to and learning from community members and incorporate their wisdom into the research project from the outset. Also, respect the process and do not rush or expect immediate results. Be patient and allow the relationship to develop naturally. Respect the contributions of community members through honorariums. By showing respect, researchers demonstrate the importance of Indigenous self-determination, and they value communities’ contributions. In turn, communities build trust that researchers will do what’s in their best interest.	Respecting community self-determination in how they set up the service transformation model, this was done through the ACCESS-OM central team traveling to be in communities to learn with and from communities and respecting community process.es
Responsibility	Be transparent about your intentions, methods and findings (challenges, setbacks) and ensure the research does no harm. Responsibility builds trust when researchers demonstrate they are accountable to communities and use research as a tool to advocate for change with communities.	Within the network, ACCESS-OM leads and staff were transparent that funding would only be available for 5 years, but that they (and the network) would work with communities to support and advocate for long term funding through evaluating the transformation within their communities through a community-based approach.
Relevancy	Ensure research is relevant to communities’ needs and priorities. It is also important to share research in relevant ways that communities identify as being meaningful; this can be a community report or developing a community billboard or a sharing circle or a visual infographic with appropriate cultural symbols. When research is relevant to communities, it shows researchers are genuinely interested in strengthening communities’ well-being.	Researchers within the network supported and mentored community members to be on peer-reviewed papers as well as funded community identified knowledge translation activities, such as a youth-directed film, billboard within community and community events.
Reciprocity	Give back to the communities in meaningful ways, this can be volunteering or participating in community events. Be consistent in your engagement, have regular and meaningful interactions over time and find ways to show that you are committed to their development. When communities feel reciprocity, they begin to trust you are working in their best interest and not the interest of the research.	The ACCESS-OM central office attended community ceremonies, Sweat Lodges, events, and launches of youth spaces.
Resources	Researchers to transfer funds to the communities to do work in meaningful ways, and be prepared to advocate for policy makers and funding decision makers to make this possible if it is not already feasible. Similarly, funders and policy makers should consider funding grant calls beyond 5-year timeframes, by recognizing that building relationships requires funding and time. Provide opportunities based on community or individual choice, such as fair compensation, and meals as well as other developmental opportunities like traveling to conferences and meetings for learning opportunities.	Our network transferred funds directly to community-partnered sites for their community service transformation and to choose which roles they would fund within their service transformation model.

## Data Availability

No datasets were generated or analysed during the current study.

## Abbreviations

IKT: Integrated knowledge translation
CBPAR: Community-based participatory action research
OCAP™: Ownership, control, access, possession
RCT: Randomized controlled trial
ACESS-OM: ACCESS open minds
